# MiR-29b reverses oxaliplatin-resistance in colorectal cancer by targeting SIRT1

**DOI:** 10.18632/oncotarget.24380

**Published:** 2018-02-01

**Authors:** Hui Liu, Xin-Hua Cheng

**Affiliations:** ^1^ Shanghai Institute of Immunology, Shanghai Jiao Tong University School of Medicine, Shanghai, 200025, China

**Keywords:** colorectal cancer, acquired drug resistance, oxaliplatin, miR-29b, SIRT1

## Abstract

Oxaliplatin is a commonly used chemotherapeutic drug for the treatment of advanced colorectal cancer. However, acquired drug resistance against oxaliplatin remains a major obstacle for efficient use of it, and mechanisms underlying oxaliplatin resistance are still required to be explored. In the present study, we exposed colorectal cancer cell line SW480 to oxaliplatin for a long time to obtain oxaliplatin-resistant colorectal cancer cell model (OR-SW480). We found that intracellular expression of miR-29b was decreased when the SW480 cells became oxaliplatin-resistant. More importantly, overexpression of miR-29b resensitized OR-SW480 cells to oxaliplatin treatment. Mechanically, gene of SIRT1 was identified to be the target of miR-29b. Overexpression of miR-29b in oxaliplatin-treated OR-SW480 decreased the expression of SIRT1 to enhance the ROS production and JNK phosphorylation, and thus promoting apoptosis via activation of caspase 9, 7 and 3. On the other hand, expression plasmid of SIRT1, N-acetyl cysteine or SP600125 (JNK specific inhibitor) abolished the effect of miR-29b on oxaliplatin-treated OR-SW480. We therefore demonstrated that miR-29b reverses oxaliplatin-resistance in colorectal cancer by targeting SIRT1/ROS/JNK pathway.

## INTRODUCTION

Colorectal cancer (CRC) is the third common malignancies, which represents the second leading cause of cancer-associated mortality worldwide [1, 2]. Although surgical therapy is the most effective treatment for CRC, patients with colorectal cancer are usually diagnosed in an advanced stage, because CRC is characterized by early metastasis and late apparent symptom [3, 4]. For these advanced CRC patients, chemotherapy is considered as the preferred treatment. However, long-term use of chemotherapeutic agents easily induces acquired drug resistance in cancers including colorectal cancer [5, 6]. Mechanisms underlying chemoresistance are required to be investigated, and strategies are urgent to be explored to increase the chemosensitivity of CRC cells.

Oxaliplatin is a commonly used chemotherapeutic drug for the treatment of advanced colorectal cancer. Oxaliplatin induces apoptosis of cancer cells by forming cross-links with DNA to inhibit DNA synthesis and replication in them [7, 8]. However, cancer cells usually develop various mechanisms such as reduced drug uptake, increased drug efflux and inactivation of reactive oxygen species (ROS) to obtain the acquired drug resistance against platinum-based chemotherapy [5, 9, 10]. Thus, numerous CRC patients have to suffer from the side effects of oxaliplatin without actually benefitting from it. Effective adjuvant therapies are required urgently to increase the chemosensitivity of CRC cells to oxaliplatin.

MicroRNAs (miRNAs) are endogenous and short non-coding RNAs, which regulate the expression of about 60% of human genes by pairing to 3′ untranslated regions (3′ UTRs) of targeted mRNAs. In addition, it is reported that over 50% of miRNAs-regulated human genes are involved in cancers [11–14]. Therefore, miRNAs are associated with cancer proliferation, metastasis and programmed cell death. Recently, studies have emphasized the association between miRNAs and chemosensitivity in colorectal cancer [15–17]. Among these miRNAs, miR-29b is reported to function as a tumor suppressor in several cancers. Absence of miR-29b is found to promote cancer cell proliferation, migration and self-renewal. Moreover, expression level of miR-29b is reported to be associated with chemosensitivity. Restore of miR-29b in cancer cells inhibit chemoresistance in some cancers [18–21]. The aim of this study is to explore the potential mechanisms underlying oxaliplatin resistance and investigate the role of miR-29b in reversing the oxaliplatin-resistance in CRC.

## RESULTS

### Overexpression of miR-29b reverses oxaliplatin-resistance in colorectal cancer cells

**Figure 1:**
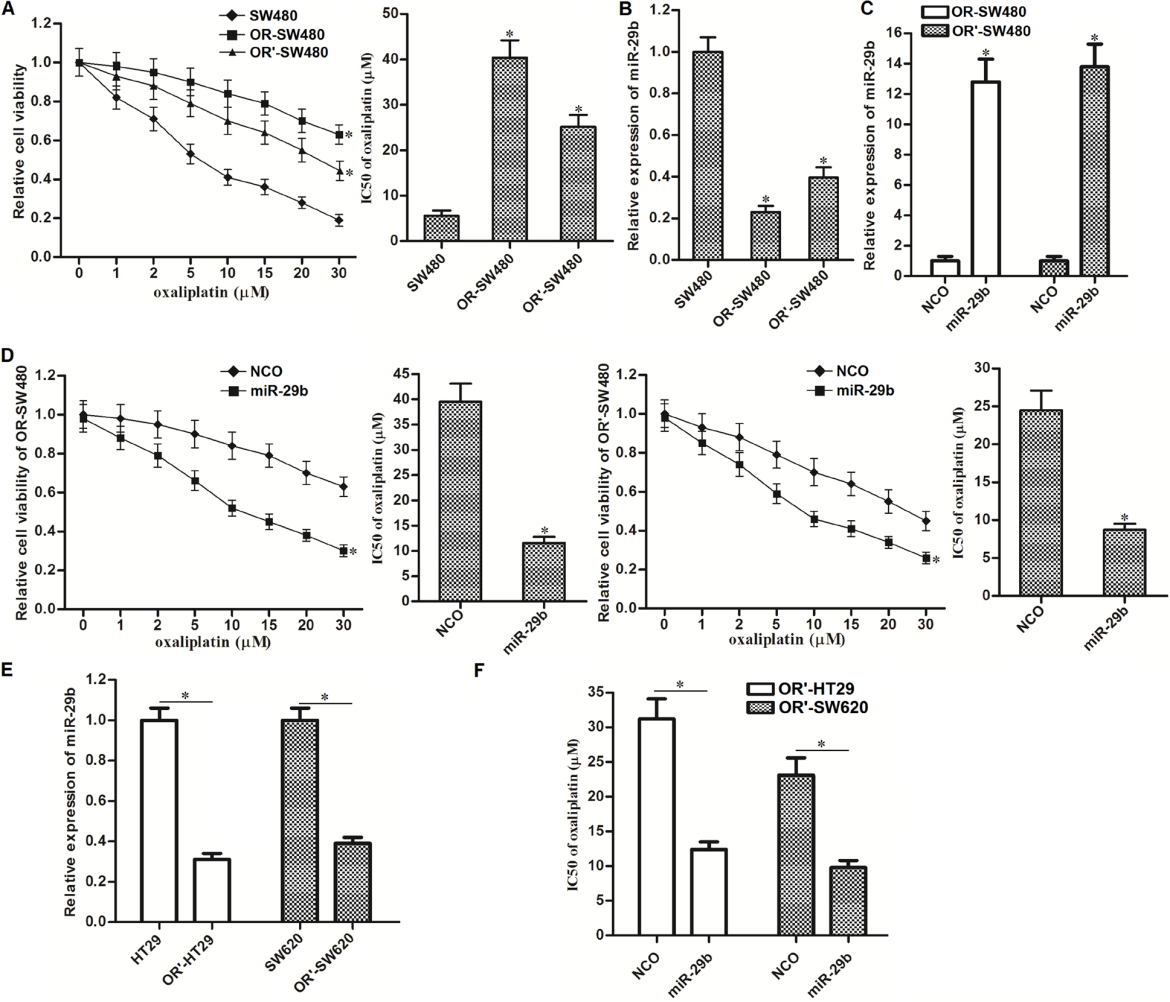
Overexpression of miR-29b reverses oxaliplatin-resistance in CRC (**A**) CCK-8 assays were performed to detect the sensitivity of SW480, OR-SW480 and OR’-SW480 to different concentrations of oxaliplatin. ^*^P < 0.05 *vs.* SW480 cells. (**B**) QRT-PCR analysis was performed to evaluate the expression of miR-29b in SW480, OR-SW480 and OR’-SW480 cells. ^*^P < 0.05 *vs.* SW480 cells. (**C**) QRT-PCR analysis was performed to evaluate the effect of miR-29b mimics on increasing the expression level of miR-29b in OR-SW480 and OR’-SW480 cells. ^*^P < 0.05 *vs.* NCO group. (**D**) CCK-8 assays were performed to evaluate the effect of miR-29b on reversing oxaliplatin-resistance in OR-SW480 and OR’-SW480. ^*^P < 0.05 *vs.* NCO group. (**E**) QRT-PCR analysis was performed to evaluate the expression of miR-29b in HT29, OR’-HT29, SW620 and OR’-SW620 cells. ^*^P < 0.05. (**F**) Effect of miR-29b on decreasing the IC50 of oxaliplatin to OR’-HT29 and OR’-SW620 to oxaliplatin. ^*^P < 0.05.

To study oxaliplatin-resistance in CRC, we exposed SW480 cells to oxaliplatin to establish the oxaliplatin-resistant colorectal cancer models (OR-SW480 for long-term exposure to oxaliplatin and OR’-SW480 for short-term exposure to oxaliplatin). Results of CCK-8 assays showed that the sensitivity of both OR-SW480 and OR’-SW480 to oxaliplatin was significantly lower than their corresponding SW480 cells. Specifically, IC50 of oxaliplatin increased by 6.2 fold to OR-SW480 3.7 fold to OR’-SW480 compared to the SW480 cells (Figure 1A). It indicated that OR-SW480 and OR’-SW480 cells were resistant to oxaliplatin. To study the role of miR-29b in oxaliplatin-resistance, we performed qRT-PCR assays. The results showed that expression of miR-29b reduced by 77.1 percent in OR-SW480 and 59.2 percent in OR’-SW480 compared to SW480 cells (Figure 1B). It suggested that downregulation of miR-29b was associated with oxaliplatin resistance in CRC. To investigate this speculation, we recovered the expression of miR-29b in OR-SW480 and OR’-SW480 by transfection with miR-29b mimics (Figure 1C). Interestingly, we observed that overexpression of miR-29b resensitized OR-SW480 and OR’-SW480 cells to oxaliplatin treatment. Specifically, IC50 of oxaliplatin reduced by 70.6 percent to OR-SW480 and 63.7 percent to OR’-SW480 because of the miR-29b transfection (Figure 1D). In addition, we also established the short-term oxaliplatin-resistant colorectal cancer models on HT29 and SW620 (OR’-HT29 and OR’-SW620, respectively). We observed significant downregulation of miR-29b in OR’-HT29 and OR’-SW620 compared to their parental cells, respectively (Figure 1E). Furthermore, overexpression of miR-29b in OR’-HT29 and OR’-SW620 was found to decrease the IC50 of oxaliplatin to them (Figure 1F). Taken together, we demonstrated that expression level of miR-29b was associated with oxaliplatin sensitivity, and overexpression of miR-29b was able to reverse the oxaliplatin-resistance in CRC.

### MiR-29b targets SIRT1 in CRC

**Figure 2:**
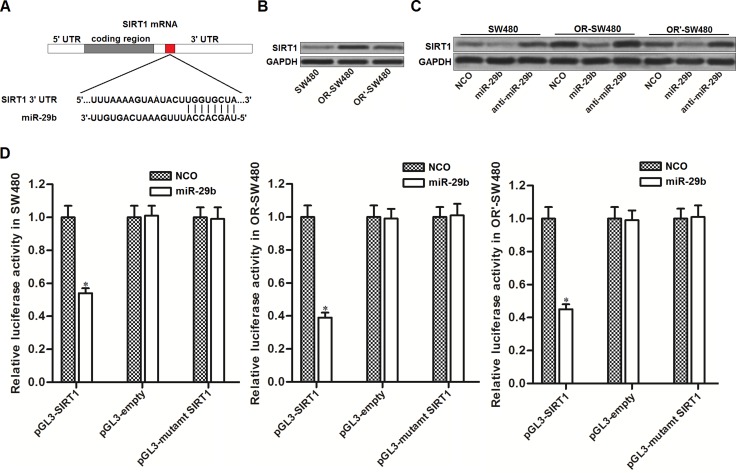
MiR-29b targets SIRT1 in CRC (**A**) Target sequences of miR-29b in the 3′ UTR of SIRT1 mRNA predicted by TargetScan database. (**B**) Western blot analysis was performed to evaluate the expression of SIRT1 in SW480, OR-SW480 and OR’-SW480 cells. (**C**) After transfection with NCO, miR-29b and anti-miR-29b, western blot analysis was performed to evaluate the role of miR-29b in changing the expression of SIRT1 in SW480, OR-SW480 and OR’-SW480 cells. (**D**) After co-transfection with miR-29b and pGL3 luciferase reporters contained SIRT1 3′ UTR, luciferase activity was determined by dual-luciferase reporter assay system in SW480, OR-SW480 and OR’-SW480 cells. ^*^P < 0.05 *vs.* NCO group.

To explore the potential mechanism by which miR-29b reversed oxaliplatin-resistance in CRC, we searched the potential target gene of miR-29b on public database called TargetScan (http://www.targetscan.org/). We found that the 3′ UTR of SIRT1 mRNA contained seed region paired with miR-29b (Figure 2A). It suggested that SIRT1 is the target of miR-29b. Results of western blot analysis showed that expression level of SIRT1 was overexpressed in OR-SW480 and OR’-SW480 cells compared to SW480 cells (Figure 2B). Since the expression of miR-29b was obviously decreased in OR-SW480 and OR’-SW480 cells compared to SW480 cells (Figure 1B), we observed negative correlation between miR-29b and SIRT1. To demonstate that miR-29b targets SIRT1 in CRC, we transfected SW480, OR-SW480 and OR’-SW480 cells with miR-29b mimics and inhibitors to evaluate its role in changing SIRT1 expression. As shown in Figure 2C, we observed that overexpression of miR-29b significantly decreased the expression of SIRT1, whereas the anti-miR-29b increased the cellular SIRT1 in all of these CRC cells. Furthermore, results of luciferase reporter assays showed that transfection with miR-29b was able to decrease the activity of luciferase reporters contained SIRT1 3′ UTR (Figure 2D). We therefore demonstrated that miR-29b targets SIRT1 in CRC. In addition, OR-SW480 exhibited lower expression level of miR-29b and higher protein level of SIRT1 compared to the OR’-SW480. Furthermore, the oxaliplatin-induced cell death in OR-SW480 can be dramatically augmented by miR-29b introduction. We therefore performed our following experiments to examine the effect of miR-29b on changing oxaliplatin-resistance to CRC in OR-SW480.

### MiR-29b reverses oxaliplatin-resistance through decreasing the expression of SIRT1 in CRC

**Figure 3:**
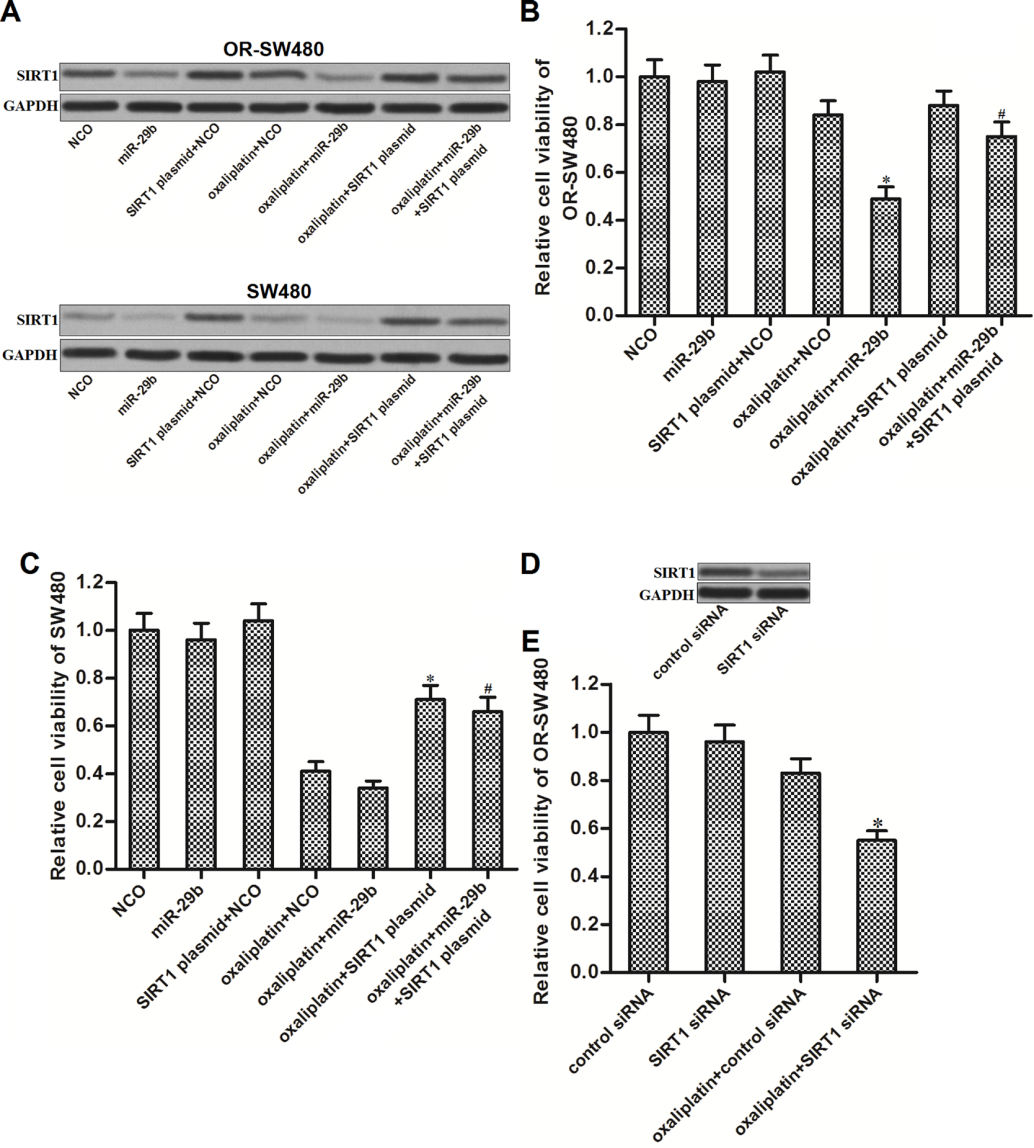
MiR-29b reverses oxaliplatin-resistance through decreasing the expression of SIRT1 in CRC (**A**) Western blot analysis was performed to evaluate the effect of oxaliplatin (10 μM), NCO, miR-29b and SIRT1 plasmid on changing the expression of SIRT1 in SW480 and OR-SW480 cells. (**B**) After treatment with oxaliplatin (10 μM), NCO, miR-29b and SIRT1 plasmid, CCK-8 assays were performed to determine the cell viability of OR-SW480 cells. ^*^P < 0.05 *vs.* oxaliplatin + NCO group, ^#^*P* < 0.05 *vs.* oxaliplatin + miR-29b group. (**C**) After treatment with oxaliplatin (10 mM), NCO, miR-29b and SIRT1 plasmid, CCK-8 assays were performed to determine the cell viability of SW480 cells. ^*^P < 0.05 *vs.* oxaliplatin + NCO group, ^#^*P* < 0.05 *vs.* oxaliplatin + miR-29b group. (**D**) Effect of SIRT1 siRNA on decreasing the protein level of SIRT1 in OR-SW480. (**E**) After treatment with oxaliplatin (10 μM), control siRNA, and SIRT1 siRNA, CCK-8 assays were performed to determine the cell viability of SW480 cells. ^*^P < 0.05 *vs.* oxaliplatin + control siRNA group.

To investigate whether miR-29b reversed oxaliplatin-resistance by targeting SIRT1 in CRC, we transfected the OR-SW480 and SW480 cells with SIRT1 plasmid to antagonize the effect of miR-29b on SIRT1 suppression. As shown in Figure 3A, transfection with SIRT1 plasmid induced overexpression of SIRT1 in SW480 and OR-SW480 even the miR-29b was co-transfected. Results of CCK-8 assays showed that although miR-29b significantly increased the sensitivity of OR-SW480 to oxaliplatin (10 μM), co-transfection with SIRT1 plasmid abolished the synergistic effect on oxaliplatin treatment (Figure 3B). In addition, oxaliplatin (10 μM) induced significant cell death of SW480. However, overexpression of SIRT1 decreased the sensitivity of SW480 cells to oxaliplatin treatment (Figure 3C). In addition, to validate that overexpression of SIRT1 was responsible for oxaliplatin-resistance in OR-SW480, we knockdown the SIRT1 expression directly by using its specific siRNA (Figure 3D). The results showed that SIRT1 siRNA significantly resensitized the OR-SW480 cells to oxaliplatin treatment similarly as the miR-29b (Figure 3E). Thus, we demonstrated that overexpression of SIRT1 was the potential mechanism for oxaliplatin-resistance. MiR-29b reverses oxaliplatin-resistance through decreasing the expression of SIRT1 in CRC.

### MiR-29b promotes oxaliplatin-induced apoptosis through the ROS pathway in OR-SW480

**Figure 4:**
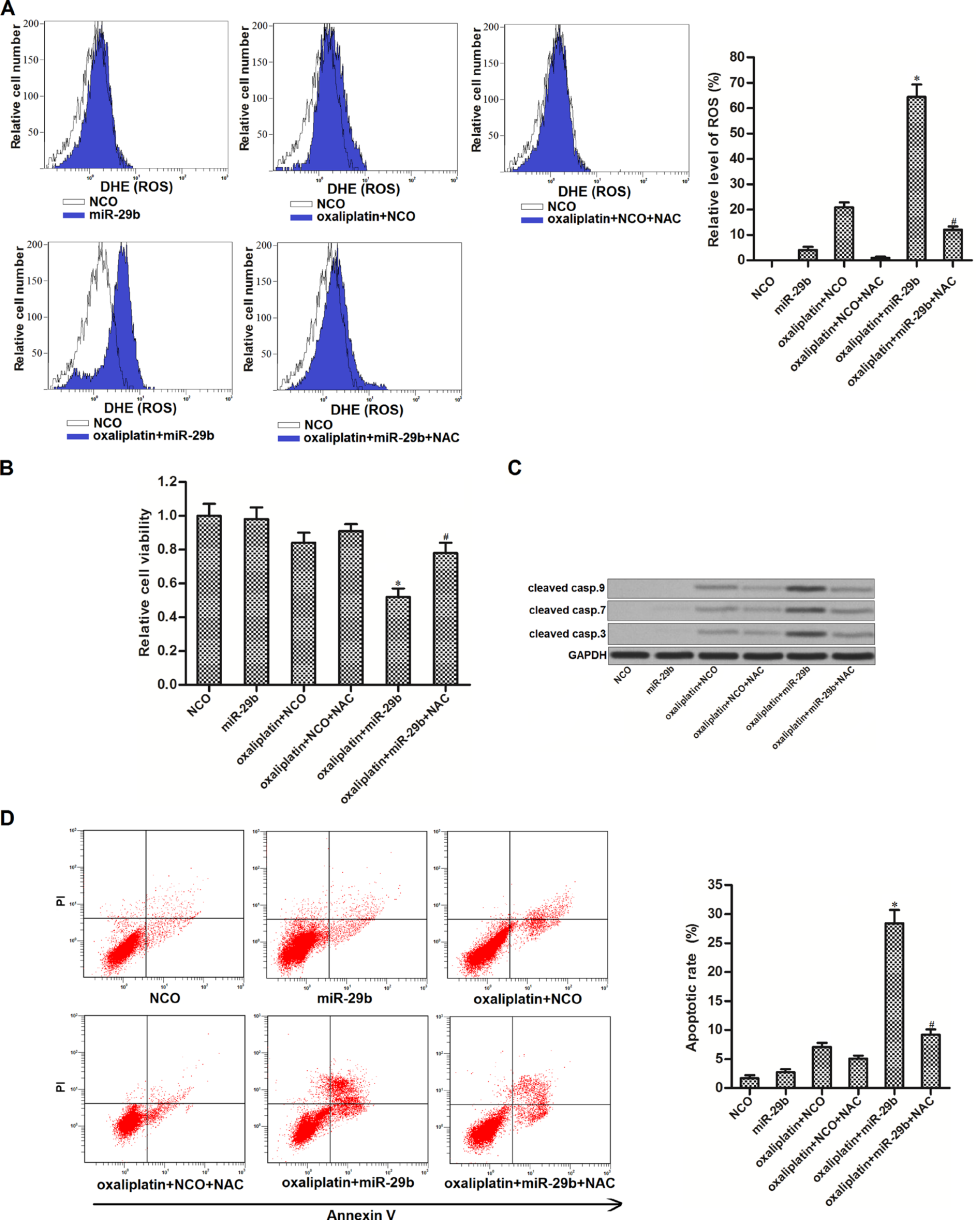
MiR-29b promoted oxaliplatin-induced apoptosis through the ROS pathway in OR-SW480 cells (**A**) After treatment with oxaliplatin (10 μM), NAC (2 mM), NCO and miR-29b, flow cytometry analysis was performed to evaluate the production of ROS in OR-SW480 cells. ^*^P < 0.05 *vs.* oxaliplatin + NCO group, ^#^*P* < 0.05 *vs.* oxaliplatin + miR-29b group. (**B**) CCK-8 assays were performed to determine the effect of NAC on cell viability of OR-SW480 cells which were co-treated with oxaliplatin (10 mM) and miR-29b. ^*^P < 0.05 *vs.* oxaliplatin + NCO group, ^#^*P* < 0.05 *vs.* oxaliplatin + miR-29b group. (**C**) Western blot analysis was performed to determine the effect of NAC on apoptosis of OR-SW480 cells which were co-treated with oxaliplatin (10 mM) and miR-29b. (**D**) Flow cytometry was performed to determine the effect of NAC on apoptosis of OR-SW480 cells which were co-treated with oxaliplatin (10 μM) and miR-29b. ^*^P < 0.05 *vs.* oxaliplatin + NCO group, ^#^*P* < 0.05 *vs.* oxaliplatin + miR-29b group.

SIRT-1 was reported to inhibit the generation of ROS in cells [22]. Thus, we speculated that overexperssion of miR-29b may promote ROS generation by decreasing the expression of SIRT-1 in OR-SW480. Results of flow cytometry analysis confirmed that although miR-29b single treatment didn’t induce significant generation of ROS in OR-SW480, it was able to promote the oxaliplatin-dependent production of ROS significantly. In addition, N-acetylcysteine (NAC), used as a ROS scavenger [23], removed the ROS induced by miR-29b and oxaliplatin co-treatment (Figure 4A). Results of CCK-8 assays showed that combination with miR-29b and oxaliplatin induced dramatic cell death of OR-SW480. However, NAC treatment significantly protected OR-SW480 cells from the cytotoxicity of them (Figure 4B). It indicated that synergistic effect of miR-29b on oxaliplatin-induced cytotoxicity to OR-SW480 was dependent on the ROS pathway. Furthermore, combination with oxaliplatin and miR-29b induced significant activation of caspase-9, -7 and -3 (Figure 4C) and occurrence of apoptosis (Figure 4D). On the other hand, NAC treatment suppressed both the caspases activation and apoptosis in oxaliplatin and miR-29b co-treated OR-SW480 cells. Together, these results demonstrated that miR-29b promoted oxaliplatin-induced apoptosis through the ROS pathway in oxaliplatin-resistant colorectal cancer cells.

### JNK is the downstream of ROS pathway in OR-SW480 cells co-treated with oxaliplatin and miR-29b

**Figure 5:**
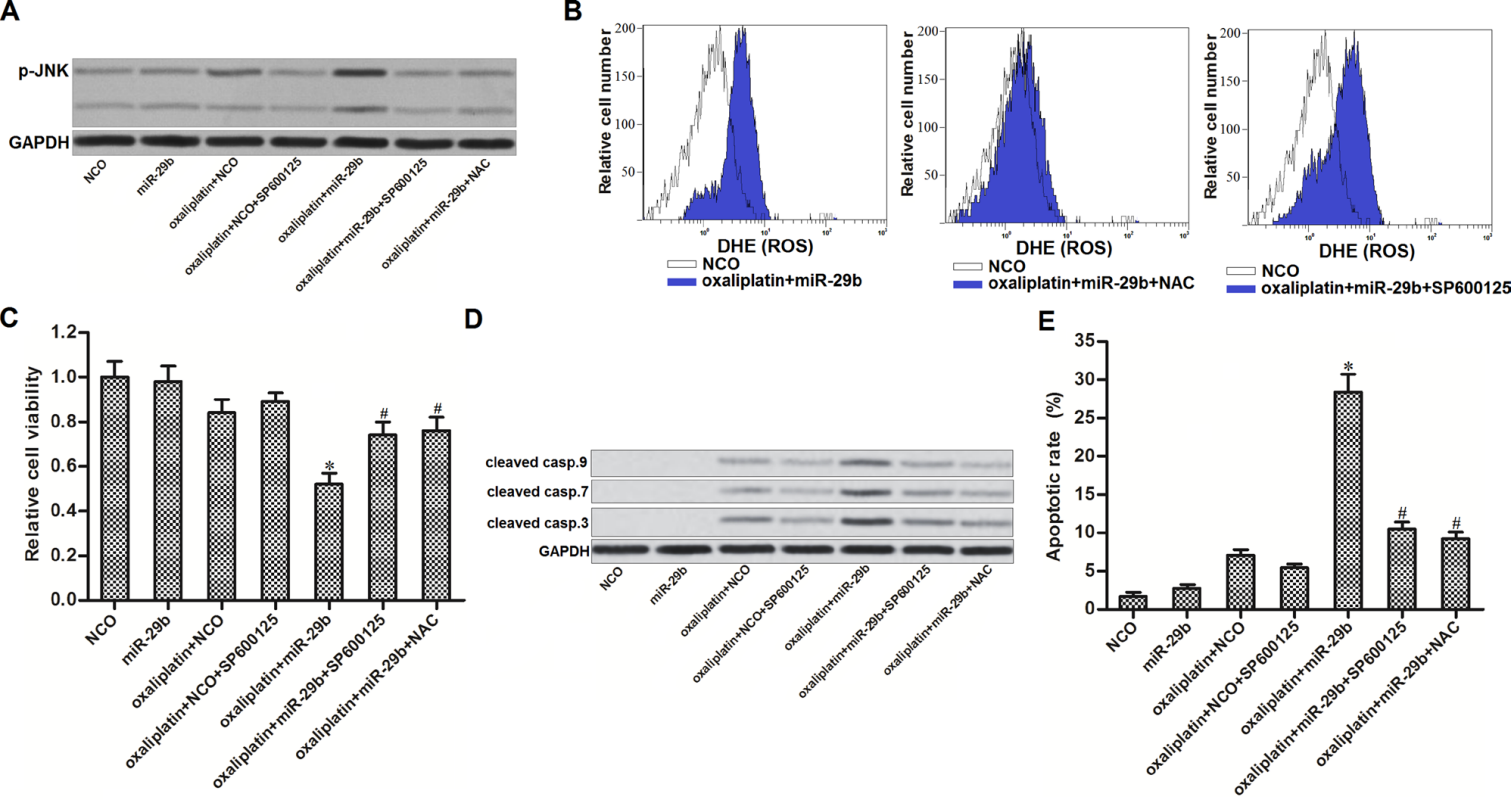
JNK is the downstream of ROS in apoptosis pathway of OR-SW480 cells co-treated with oxaliplatin and miR-29b (**A**) After treatment with oxaliplatin (10 μM), SP600125 (50 μM), NAC (2 mM), NCO and miR-29b, western blot analysis was performed to evaluate the phosphorylation of JNK in OR-SW480 cells. (**B**) Flow cytometry analysis was performed to determine the effect of SP600125 (50 μM) and NAC (2 mM) on ROS production. (**C**) CCK-8 analysis was performed to determine the effect of SP600125 (50 μM) and NAC (2 mM) on cell viability of OR-SW480 cells co-treated with oxaliplatin (10 μM) and miR-29b. ^*^P < 0.05 *vs.* oxaliplatin + NCO group, ^#^*P* < 0.05 *vs.* oxaliplatin + miR-29b group. (**D**) Western blot analysis was performed to evaluate the activation of caspase-9, -7 and -3 after the OR-SW480 cells were treated with oxaliplatin (10 μM), SP600125 (50 μM), NAC (2 mM), NCO and miR-29b. (**E**) Apoptotic rate of OR-SW480 was measured by flow cytometry analysis after they were treated with oxaliplatin (10 μM), SP600125 (50 μM), NAC (2 mM), NCO and miR-29b. ^*^P < 0.05 *vs.* oxaliplatin + NCO group, ^#^*P* < 0.05 *vs.* oxaliplatin + miR-29b group.

Previous studies indicated that JNK pathway is a molecular linkage between oxidative stress and cellular apoptosis [24]. Thus, we next investigated the JNK pathway in OR-SW480 cells co-treated with oxaliplatin and miR-29b. As shown in (Figure 5A), oxaliplatin-induced phosphorylation of JNK can be significantly enhanced by miR-29b co-treatment. SP600125, a JNK specific inhibitor [25], was tested to inhibit the phosphorylation of JNK induced by combination with oxaliplatin and miR-29b in OR-SW480 cells. Interestingly, we observed that NAC treatment also suppressed the phosphorylation of JNK in oxaliplatin and miR-29b co-treated OR-SW480 cells. On the other hand, results of flow cytometry analysis showed that SP600125 treatment can not reduce the ROS production induced by combination with oxaliplatin and miR-29b (Figure 5B). Together, these results indicated that combination with oxaliplatin and miR-29b induced activation of JNK, which was the downstream of ROS pathway. To investigate the role of ROS/JNK pathway in cell viability and apoptosis of OR-SW480, several experiments were performed. Results of CCK-8 assays showed that both SP600125 and NAC increased the cell viability of OR-SW480 cells which were co-treated with oxaliplatin and miR-29b (Figure 5C). Furthermore, results of western blot and flow cytometry analysis showed that either SP600125 or NAC was able to reduce the activation of caspase-9, -7 and -3 (Figure 5D) and occurrence of apoptosis (Figure 5E). We thus demonstrated that overexperssion of miR-29b was able to promote oxaliplatin-induced apoptosis through the ROS/JNK pathway in oxaliplatin-resistant colorectal cancer cells.

### Overexpression of miR-29b reverses oxaliplatin-resistance *in vivo*

**Figure 6:**
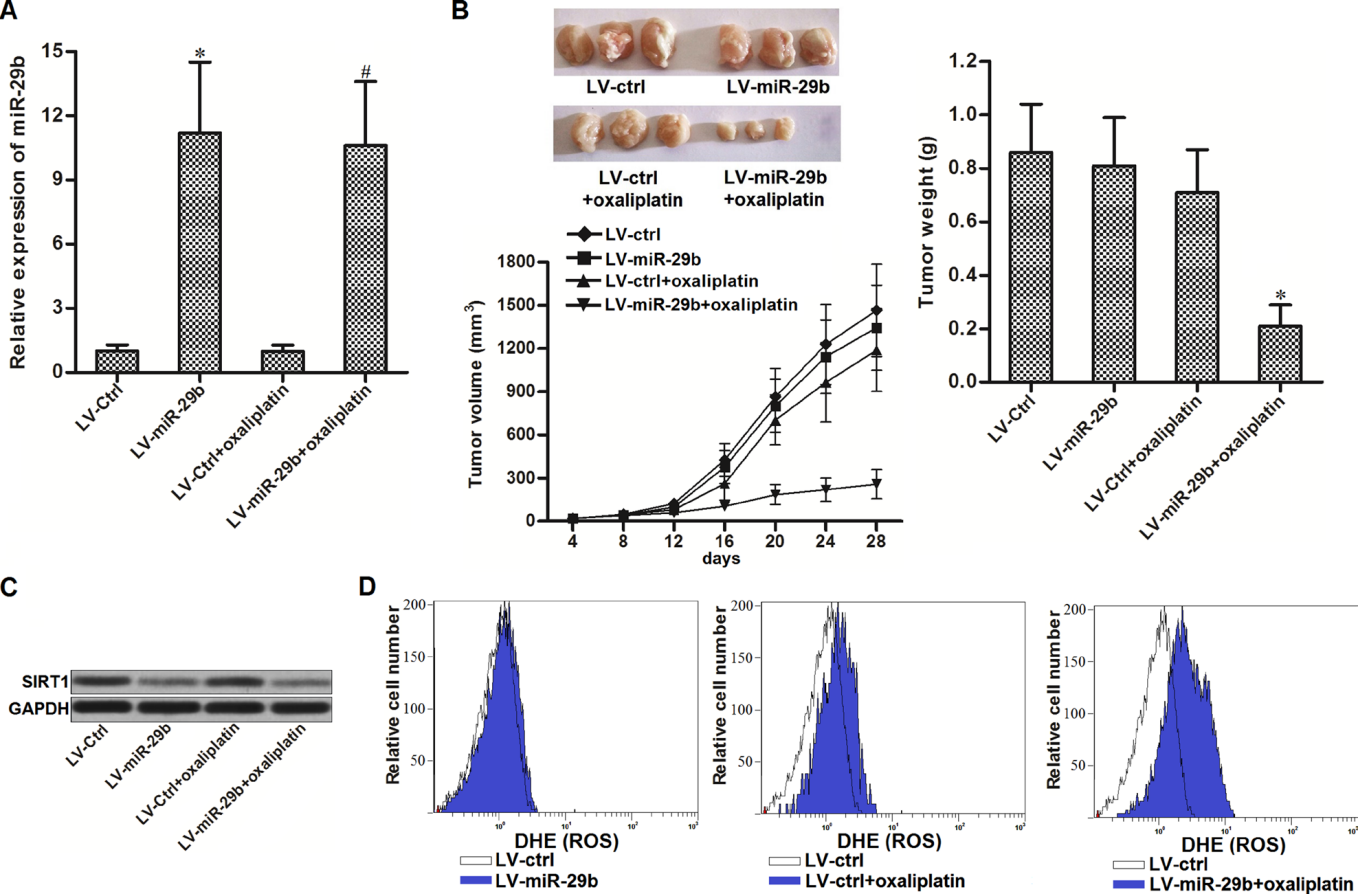
Overexpression of miR-29b reversed oxaliplatin-resistance *in vivo* (**A**) Expression of miR-29b in tumor tissues was measured by qRT-PCR analysis. ^*^P < 0.05 *vs.* LV-ctrl group, ^#^*P* < 0.05 *vs.* LV-ctrl + oxaliplatin group. (**B**) Effect of miR-29b and oxaliplatin on tumor volume and weight of OR-SW480 originated tumors. ^*^P < 0.05 *vs.* LV-ctrl + oxaliplatin group. (**C**) Western blot analysis was performed to evaluate the expression of SIRT1 in purified tumor tissue cells. (**D**) Flow cytometry analysis was performed to detect the ROS generation in purified tumor tissue cells.

To investigate the *in vivo* effect of miR-29b on oxaliplatin-resistance in CRC, we established colorectal cancer model in mice by using oxaliplatin-resistant SW480 cells transduced with LV-miR-29b or LV-ctrl. 28 days after inoculation with OR-SW480 cells, mice were sacrificed and miR-29b expression in tumor tissues was measured. As shown in (Figure 6A), expression of miR-29b was higher in the LV-miR-29b groups than that in the LV-ctrl groups. Before sacrifice, the mice were treated with oxaliplatin (10 mg/kg body weight) twice a week. We observed that the tumors originated from LV-ctrl-transduced OR-SW480 were resistant to oxaliplatin treatment. In contrast, miR-29b-overexpressed tumors exhibited higher sensitivity to oxaliplatin treatment rather than the control ones (Figure 6B). In the purified cells from the separated tumor tissues, we found that the expression level of SIRT1 in LV-miR-29b groups was obviously lower than that in the LV-ctrl groups (Figure 6C). Furthermore, we observed that the LV-miR-29b-transduced tumor cells were easier to generate ROS compared to the LV-ctrl-transduced ones after the oxaliplatin treatment (Figure 6D). These results suggested that overexpression of miR-29b in CRC was able to reverse the oxaliplatin-resistance through the SIRT1/ROS pathway *in vivo*.

### Overexpression of miR-29b reverses cross-resistance of OR-SW480 cells to cisplatin and carboplatin

**Figure 7:**
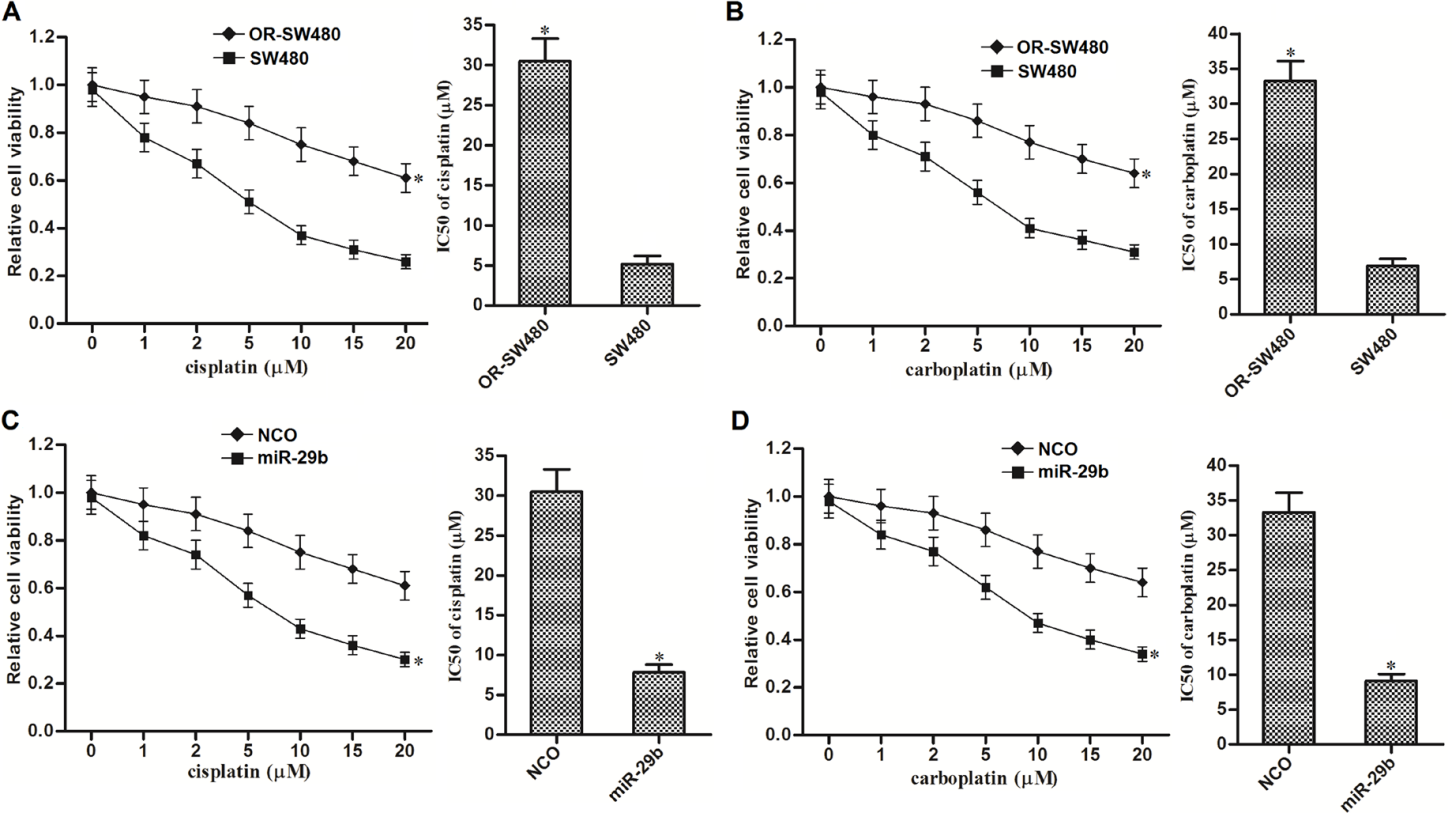
MiR-29b reversed cross-resistance of OR-SW480 cells to platinum-based treatment (**A**) Sensitivity of OR-SW480 to cisplatin treatment. ^*^P < 0.05 *vs.* SW480 cells. (**B**) Sensitivity of OR-SW480 to carboplatin treatment. ^*^P < 0.05 *vs.* SW480 cells. (**C**) Effect of miR-29b on reversing cisplatin-resistance to OR-SW480. ^*^P < 0.05 *vs.* SW480 cells. (**D**) Effect of miR-29b on reversing carboplatin-resistance to OR-SW480. ^*^P < 0.05 *vs.* SW480 cells.

Compared to SW480 cells, IC50 of cisplatin to OR-SW480 cells increased 4.9 fold (Figure 7A) and IC50 of carboplatin to OR-SW480 cells increased 3.8 fold (Figure 7B). It indicated that OR-SW480 cells exhibited significant cross-resistance to platinum-based therapy. However, we observed that miR-29b co-treatment significantly increased the sensitivity of OR-SW480 cells to cisplatin (Figure 7C) and carboplatin (Figure 7D). We therefore demonstrated that overexpression of miR-29b was able to reverse the cross-resistance of oxaliplatin-resistant CRC cells to platinum-based treatment.

## DISCUSSION

Oxaliplatin is the third-generation of platinum-based drug. Nowadays, it is widely used as one best treatment option in digestive cancers including gastric and colorectal cancer [26, 27]. However, high proportion (50–70%) of CRC patients suffered from the innate and acquired resistance to chemotherapy [28]. Therefore, combination with adjuvant agents in oxaliplatin-based chemotherapy is required to overcome the major clinical problem of drug-resistance.

Studies have demonstrated that change of miRNA profile induces chemoresistance in human cancers including CRC. Correcting the dysregulation of target miRNAs is considered as a potential strategy to solve the problem of chemoresistance [16, 17, 29, 30]. In the present study, we revealed that miR-29b expression was significantly reduced in established oxaliplatin-resistant CRC cells. More importantly, our results of CCK-8 assays showed that miR-29b has a close association with drug-resistance of CRC cells. Overexpression of miR-29b recovered the sensitivity of oxaliplatin-resistant CRC cells to oxaliplatin treatment. Therefore, Adjuvant therapy of miR-29b may represent a novel strategy against the chemoresistance in CRC.

Next we investigated the mechanism by which miR-29b reversed the oxaliplatin-resistance in CRC. Among the predicted targets of miR-29b such as signal transducer and activator of transcription 3 (Stat3), cyclin-dependent kinase 6 (CDK6), lysine demethylase 2A (KDM2A) and Sirtuin-1 (SIRT-1), SIRT-1 was reported to be associated with chemoresistance in cancers [31, 32, 33]. Furthermore, our data of preliminary experiments showed that expression level of SIRT-1 was dramatically upregulated in the oxaliplatin-resistant CRC cells. We therefore focused on SIRT-1 for the further study.

SIRT-1 belongs to the family of histone deacetylase protein. Studies have demonstrated that SIRT-1 participated in the oncogenic signaling pathway of several cancers. Overexpression of SIRT-1 was found to promote proliferation and metastasis in osteosarcoma, pancreatic cancer, lung cancer and colorectal cancer [34, 35, 36, 37]. High level of SIRT-1 usually implicated poor prognosis in patients with cancer [38, 39]. Moreover, Overexpression of SIRT-1 was reported to contribute to chemoresistance in several cancers. Knockdown of SIRT-1 was found to be able to increase the sensitivity of cancer cells to chemotherapy [31, 32, 33]. These reports indicate that SIRT-1 is a potential target for improving chemosensitivity.

SIRT-1 functions as a regulator of cellular antioxidant. Overexpression of SIRT-1 increased the expression of cellular antioxidants such as superoxide dismutase (SOD), thereby eliminating the cellular ROS to evade the mitochondrial apoptosis [18, 40, 41]. In ROS-dependent pathway of apoptosis, JNK is one of the important downstream molecular linkage between oxidative stress and cellular apoptosis [20]. Activation of JNK increases the expression of pro-apoptotic proteins and block the function of Bcl-2 which is an important anti-apoptotic protein [42–44]. These studies suggest that overexpression of SIRT-1 is responsible for anti-apoptosis by suppressing the ROS/JNK pathway.

Generation of ROS participates in the mitochondrial apoptosis induced by platinum-based chemotherapy [9, 45, 46]. In the present study, our results showed that oxaliplatin-resistant CRC cells significantly overexpressed SIRT-1. High level of SIRT-1 prevented the production of ROS induced by oxaliplatin treatment. Therefore, oxaliplatin-resistant CRC cells exhibited low response to mitochondrial apoptosis. However, we found that SIRT-1 was the target of miR-29b. Overexpression of miR-29b was observed to promote the oxaliplatin-dependent generation of ROS through suppressing the expression of SIRT-1. Both SP600125 and NAC inhibited the synergistic effect of miR-29b on oxaliplatin-induced activation of caspases and apoptosis. These results demonstrated that overexpression of miR-29b reversed oxaliplatin-resistance through the SIRT-1/ROS/JNK pathway in colorectal cancer.

In summary, we demonstrated that miR-29b treatment can reverse the resistance of CRC cells to oxaliplatin therapy. Furthermore, we also provided evidence that cross-resistance of oxaliplatin-resistant CRC cells to other platinum-based chemotherapeutic agents such as cisplatin and carboplatin can be reduced by combination with miR-29b. These findings suggest a novel strategy of miRNA adjuvant treatment to improve the efficiency of platinum-based chemotherapy in CRC.

## MATERIALS AND METHODS

### Cell lines

Human CRC cell line SW480, HT29 and SW620 was purchased from American Type Culture Collection (ATCC, Rockville, MD, USA). Oxaliplatin-resistant CRC cells were obtained by continuous exposure to oxaliplatin. For short-term exposure to oxaliplatin, SW480, HT29 and SW620 CRC cells were treated with 0.5 mM oxaliplatin for 1 month, and they were named as OR’-SW480, OR’-HT29 and OR’-SW620. For long-term exposure to oxaliplatin, SW480 cells were initially treated with 0.5 mM oxaliplatin. 11 months later, SW480 cells were finally cultured in 2.5 mM oxaliplatin (oxaliplatin concentration was increased every 1 month by 0.2 μM). These oxaliplatin-induced SW480 cells exhibited more significant oxaliplatin-resistance compared to the OR’-SW480, and they were named OR-SW480. All of the above cells were cultured in Dulbecco’s modified Eagle’s medium (DMEM) containing 10% fetal bovine serum.

### miRNA and plasmid transfection

Mature human miR-29b (5′-UAGCACCAUU UGAAAUCAGUGUU-3′), negative control oligon ucleotides (NCO, 5′-AUAUAGUCAUGUUCCAC UAGUG-3′), 2’-Omethyl modified miR-29b inhibitors (anti-miR-29b, 5′-AACACUGA UUUCAAAUGGUGCUA-3′) and SIRT1 siRNA (forward: 5′-GAUGACGUCUUAUCCUCUAUU-3′, reverse: 5′-UAGAGGAUAAGACGUCAUCUU-3′) were purchased from RiboBio Co. Ltd (Guangzhou, China). For overexpression of SIRT1, open reading frame region of human SIRT1 was reconstructed with pcDNA3.1 eukaryotic expression plasmid (Invitrogen, USA). For transfection, 50 pmol/ml miR-29b, 50 pmol/ml anti-miR-29b, 50 pmol/ml NCO, 50 pmol/ml SIRT1 siRNA or 2 μg/ml SIRT1 plasmid was transfected into CRC cells by using Lipofectamine 2000 (Invitrogen) according to the manufacturer’s instructions.

### Quantitative real-time polymerase chain reaction (qRT-PCR) for miR-29b detection

Total RNA of CRC cells was extracted with TRIzol reagent (Invitrogen). RNAs were reversely transcribed by One Step PrimeScript miRNA cDNA Synthesis Kit (TaKaRa, China) according to the manufacturer’s instructions. Expression of miR-29b was detected by qRT-PCR with SYBR Premix Ex Taq (TaKaRa) on the Applied Biosystems 7500 Sequence Detection system (Applied Biosystems, USA). MiR-29b primer sequence is as follows: 5′-TAGCACCATTTGAAATCAGTGTT-3′. U6 snRNA was chosen as the endogenous control to normalize the relative expression of miR-29b.

### Drug sensitivity analysis

After transfection with miR-29b, NCO and SIRT1 plasmid, 5 × 10^3^ CRC cells were seeded on 96-well plates and treated with chemotherapeutic drugs for 48 h. Subsequently, 10 μl CCK-8 solution was added to the cells followed by incubating at 37°C for additional 2 h. Absorbance at 450 nm was measured by using an ELISA microplate reader. Half maximal inhibitory concentration (IC50) of chemotherapeutic drugs was calculated according to the cell viability of SW480 and OR-SW480 cells.

### Western blot analysis

After transfection with miR-29b, NCO and SIRT1 plasmid, OR-SW480 cells were treated with oxaliplatin (10 μM) for 48 h. Whole cell lysates were then prepared by using RIPA buffer (Cell Signaling Technology, Beverly, USA). Equal amount of protein samples were then separated by 10% sodium dodecyl sulfate-polyacrylamide gel electrophoresis (SDS-PAGE) followed by transferring to polyvinylidene fluoride (PVDF) membranes (Millipore, Billerica, MA, USA). The membranes first blocked with 5% skim milk and incubated with the primary antibodies of SIRT1 (#2496, cell signaling technology, USA), cleaved caspase 9 (#20750, cell signaling technology), cleaved caspase 7 (#8438, cell signaling technology), cleaved caspase 3 (#9664, cell signaling technology), Phospho-JNK (#9254, cell signaling technology) and GAPDH (#5174, cell signaling technology). After incubation with appropriate secondary antibodies, the protein bands were detected by using an enhanced chemiluminescent substrate (Thermo Fisher Scientific, Inc, USA).

### Luciferase reporter assay

Human SIRT1 3′ UTR contained with seed region of miR-29b was cloned into pGL3 luciferase reporter vector (Promega, USA) downstream of the luciferase gene. The primer sequences are as follows: forward: 5′-GGGGTACCATAATTTTTAACTTCATTAT-3′, reverse: 5′-GAAGATCTTATTACAAGTTACATCAT CAT-3′. For luciferase reporter assay, 50 pmol/ml miR-29b, 2 μg/ml recombinant pGL3 luciferase reporter vector and 100 ng/ml renilla luciferase pRL-TK vector (Promega) were co-transfected into SW480, OR-SW480 and OR’-SW480 cells. 24 h after transfection, cells were collected and lysed. Activities of pGL3-firefly luciferase and pRL-TK-renilla luciferase were analyzed by using dual-luciferase reporter assay system (Promega) according to the manufacturer’s protocol. Firefly luciferase activities were normalized to renilla luciferase activities

### Detection of apoptosis and ROS

After transfection with miR-29b, NCO and SIRT1 plasmid, OR-SW480 cells were treated with oxaliplatin (10 μM) for 48 h. Subsequently, cells were collected and washed with PBS. For apoptosis measurement, cells were incubated with Annexin V and PI (Sigma Aldrich, USA) followed by analyzing on flow cytometry. For evaluation of ROS production, cells were stained with dihydroethidium (DHE) (Molecular Probes, USA) followed by analyzing on flow cytometry. The non-overlapping area normalized to the NCO group was represented as the generated ROS in the experimental groups.

### Tumor growth in nude mice

Before animal experiments, OR-SW480 cells were transfected with recombinant lentivirus contained miR-29b precusor sequence (50 transducing units per cell) (Shanghai Genechem Co., Ltd., Shanghai, China) and selectively cultured in 1 μg/ml puromycin for 2 weeks. 5 × 10^6^ lentivirus-miR-29b (LV-miR-29b) or empty lentivirus (LV-control) transfected OR-SW480 cells were injected into dorsal skin of four-week-old immunodefcient nude BALB/c mice (Shanghai Super-B&K Laboratory Animal Corp., Ltd., Shanghai, China). Oxaliplatin (10 mg/kg body weight) was administrated by intraperitoneal injection twice a week. Tumor size was measured every 4 days. 28 days post-injection, the tumors were taken out and their weight were recorded after the mice were sacrificed. For detection of miR-29b, SIRT1 expression and ROS generation in tumor tissues, collagenase type III was used to purify them. The animal care and experimental protocols were approved by the Animal Care Committee of Shanghai Jiao Tong University School of Medicine.

### Statistical analysis

All the experiments were independently repeated at least 3 times. Data were analyzed by using SPSS 14.0. Two-sided Student’s *t*-test was used to estimate the statistical differences between two groups. One-way analysis of varianve (ANOVA) was used to determine the differences between three or more groups. Differences were considered statistically significant when *P* values < 0.05.
